# Structural Basis for Ubiquitin Recognition by Ubiquitin-Binding Zinc Finger of FAAP20

**DOI:** 10.1371/journal.pone.0120887

**Published:** 2015-03-23

**Authors:** Aya Toma, Diane T. Takahashi, Yusuke Sato, Atsushi Yamagata, Sakurako Goto-Ito, Shinichiro Nakada, Atsuhiko Fukuto, Yasunori Horikoshi, Satoshi Tashiro, Shuya Fukai

**Affiliations:** 1 Structural Biology Laboratory, Life Science Division, Synchrotron Radiation Research Organization and Institute of Molecular and Cellular Biosciences, The University of Tokyo, Tokyo, 113-0032, Japan; 2 Department of Medical Genome Sciences, Graduate School of Frontier Sciences, The University of Tokyo, Chiba, 277-8501, Japan; 3 Department of Bioregulation and Cellular Response, Graduate School of Medicine, Osaka University, Osaka, 565-0871, Japan; 4 Department of Cellular Biology, Research Institute for Radiation Biology and Medicine, Hiroshima University, Hiroshima, 734-8553, Japan; Hokkaido University, JAPAN

## Abstract

Several ubiquitin-binding zinc fingers (UBZs) have been reported to preferentially bind K63-linked ubiquitin chains. In particular, the UBZ domain of FAAP20 (FAAP20-UBZ), a member of the Fanconi anemia core complex, seems to recognize K63-linked ubiquitin chains, in order to recruit the complex to DNA interstrand crosslinks and mediate DNA repair. By contrast, it is reported that the attachment of a single ubiquitin to Rev1, a translesion DNA polymerase, increases binding of Rev1 to FAAP20. To clarify the specificity of FAAP20-UBZ, we determined the crystal structure of FAAP20-UBZ in complex with K63-linked diubiquitin at 1.9 Å resolution. In this structure, FAAP20-UBZ interacts only with one of the two ubiquitin moieties. Consistently, binding assays using surface plasmon resonance spectrometry showed that FAAP20-UBZ binds ubiquitin and M1-, K48- and K63-linked diubiquitin chains with similar affinities. Residues in the vicinity of Ala168 within the α-helix and the C-terminal Trp180 interact with the canonical Ile44-centered hydrophobic patch of ubiquitin. Asp164 within the α-helix and the C-terminal loop mediate a hydrogen bond network, which reinforces ubiquitin-binding of FAAP20-UBZ. Mutations of the ubiquitin-interacting residues disrupted binding to ubiquitin *in vitro* and abolished the accumulation of FAAP20 to DNA damage sites *in vivo*. Finally, structural comparison among FAAP20-UBZ, WRNIP1-UBZ and RAD18-UBZ revealed distinct modes of ubiquitin binding. UBZ family proteins could be divided into at least three classes, according to their ubiquitin-binding modes.

## Introduction

Modification of proteins by ubiquitin (Ub) conjugation (ubiquitylation) regulates protein degradation, activity, localization and interactions and thus is involved in most cellular processes [1]. Proteins can be attached to a single Ub, or to a Ub chain, where the C-terminal glycine residue of each Ub is linked to the N-terminal methionine residue or one of the seven lysine residues of the following Ub. Ubiquitylated targets promote different cellular signaling, depending on the chain length and/or linkage between Ub moieties. This feature primarily relies on the recognition of Ub by Ub-binding domains (UBDs). To date, more than twenty classes of UBDs have been identified [2]. Several UBDs recognize Ub chains with specificity towards the linkage between Ub moieties. For example, Npl4 zinc finger (NZF) of TAB2/3, tandem Ub-interacting motif (tUIM) of RAP80 and CARD domain of RIG-I specifically bind K63-linked Ub chains (K63 chains), while HOIL-1L NZF, A20 zinc finger 7 and UBAN domain of NEMO specifically bind M1-linked Ub chains (M1 chains). Three-dimensional structures of these UBDs in complex with their cognate Ub chains have provided important insights into mechanisms for the linkage specificities [3–9].

Recently, Ub-binding zinc fingers (UBZs) have emerged as potential K63-linkage-specific UBDs. Five publications reported that UBZs of RAD18, WRNIP1, FAAP20 and SLX4 bind specifically K63 chains [10–14], unlike those of Polη, Polκ, and FAN1, which seem to bind mono-ubiquitylated targets [15–18]. The interaction between UBZ and Ub or Ub chains plays important roles in the Fanconi anemia (FA) pathway, which repairs DNA interstrand crosslinks (ICLs) and other replication blocking DNA damage. Upon ICL formation, RNF8 together with UBC13-MMS2 or UBC13-UEV1a rapidly assembles K63 chains on histone H2A and other targets [11]. Several publications indicate that K63 chains are recognized by the UBZ domain of FAAP20 (FAAP20-UBZ), which recruits the FA core complex (FANCA, FANCB, FANCC, FANCE, FANCF, FANCG, FANCL, FANCM, FAAP20, FAAP24 and FAAP100) through its interaction with FANCA [11–13]. Within the FA core complex, FANCL mono-ubiquitylates FANCI/FANCD2 [19]. This activity relies on RAD6/RAD18 ubiquitylation of PCNA [20]. The E3 ligase RAD18 itself possesses a UBZ domain, which seems important for recruitment of RAD6/RAD18 to DNA damage sites [21]. Thereafter, nucleases are recruited to cleave off damaged DNA. Notably, FAN1 and SLX4 nucleases possess UBZ domains. The UBZ domain of FAN1 binds mono-ubiquitylated FANCI/FANCD2 [22–24]. On the other hand, the SLX4 UBZ domain seems to bind K63 chains, although its precise target is still unidentified [14]. The gap left by nucleases is filled using specialized polymerase, including Polν, Polζ and REV1. Recently, REV1 was shown to be ubiquitylated and this ubiquitylation may trigger the recruitment of FAAP20 [25]. However, this function contradicts other papers, because mono-ubiquitylation of REV1 seems sufficient to recruit FAAP20, suggesting that FAAP20-UBZ can bind Ub as well as Ub chains *in vivo*. The linkage specificity of FAAP20-UBZ is still controversial.

In this study, to clarify the specificity of FAAP20-UBZ towards Ub and Ub chains, we solved the crystal structure of FAAP20-UBZ in complex with K63-linked Ub_2_ at 1.9 Å resolution. The present complex structure and surface plasmon resonance (SPR) spectrometric analysis showed that FAAP20-UBZ binds Ub and K63-, M1- and K48-linked Ub_2_ species with similar affinities. FAAP20-UBZ structure lacks the anti-parallel β sheets and differs from the canonical ββα fold found in the other UBZ structures identified so far [26–29]. FAAP20-UBZ contains an α-helix reminiscent of other Ub-binding domain, the following Ub-binding loop and a stabilizing loop at the N-terminal extremity. Site-directed mutational analyses revealed that Ub-interacting residues of FAAP20-UBZ are critical for binding to Ub *in vitro* and accumulation of FAAP20 to ICL sites *in vivo*.

## Materials and Methods

### Preparation of FAAP20-UBZ

The gene encoding human FAAP20-UBZ (residues 142–180) was PCR amplified from a human cDNA library. The amplified gene was cloned into the pCold-GST expression vector with *Nde*I and *Xho*I sites to produce N-terminal GST fusion protein and confirmed by DNA sequencing. *E*. *coli* strain Rosetta (DE3) cells (Invitrogen) were transformed with the expression vector and cultured in LB media containing 100 mg/L ampicillin at 37°C. When the optical density at 600 nm of the culture reached ~0.5, isopropyl-β-d-thiogalactopyranoside (IPTG) was added to a final concentration of 0.3 mM to induce protein expression for 24 h at 15°C. The cells were collected by centrifugation at 7,000 *g* for 15 minutes and disrupted by sonication in phosphate buffered saline (PBS) containing 1 mM dithiothreitol (DTT) and 0.5% Triton X-100. The lysates were centrifuged at 28,000 *g* for 60 minutes and the supernatants were then loaded onto a Glutathione Sepharose FF (Qiagen) column that had been pre-equilibrated with PBS containing 1 mM DTT and 0.5% Triton X-100. The column was washed with the PBS containing 1 mM DTT and 0.5% Triton X-100 and then with PBS containing 1 mM DTT. GST fusion samples were eluted with 50 mM Tris-HCl buffer (pH 8.0) containing 200 mM NaCl, 1 mM DTT and 15 mM reduced glutathione. The samples were dialyzed against 50 mM Tris-HCl buffer (pH 8.0) containing 1 mM NaCl and 1 mM DTT. The proteins were loaded onto a ResourceQ anion exchange column (GE Healthcare) pre-equilibrated with 50 mM Tris-HCl buffer (pH 8.0) containing 1 mM DTT and were eluted with a linear gradient of 0-1M NaCl. The peak fractions containing FAAP20-UBZ were collected, and the GST-tags were cleaved by HRV3C protease at 4°C. The samples were loaded onto a HiLoad 16/60 Superdex75 (prep grade) column (GE Healthcare) pre-equilibrated with 10 mM Tris-HCl buffer (pH 7.2) containing 50 mM NaCl and 5 mM β-mercaptoethanol. The peak fractions containing purified FAAP20-UBZ were collected.

### Crystallization and data collection

To prepare FAAP20-UBZ•K63-Ub_2_ complex, a 1.5-fold molar excess of FAAP20-UBZ was incubated at 4°C overnight with K63-Ub_2_ that was prepared as described previously [30]. The FAAP20-UBZ•K63-Ub_2_ complex was loaded onto a HiLoad 16/60 Superdex75 (prep grade) column (GE Healthcare) pre-equilibrated with 10 mM Tris-HCl buffer (pH 7.2) containing 50 mM NaCl and 5 mM β-mercaptoethanol, to remove unbound K63-Ub_2_. Purified FAAP20-UBZ•K63-Ub_2_ complex was concentrated to ~10 g/L, by using an Amicon Ultra-4 3,000 MWCO filter (Millipore), following the manufacturer’s instructions. Initial crystallization screening was performed using the sitting drop vapor diffusion method at 20°C, with a Mosquito liquid-handling robot (TTP Lab Tech). We tested about 400 conditions, using crystallization reagent kits supplied by Hampton Research, and initial hits were further optimized. The best crystals of the FAAP20-UBZ•K63-Ub_2_ complex grew at 20°C with the sitting drop vapor diffusion method by mixing 0.5 μl of a protein solution with an equal amount of a precipitant solution containing 1.8 M DL-malic acid (pH 7.0), 0.1 M glycine. For data collection, the crystals of the FAAP20-UBZ•K63-Ub_2_ complex were transferred to the precipitant solution containing saturated trehalose for cryoprotection. Cryoprotected crystals were flash frozen in liquid nitrogen.

### Structural determination and refinement

Diffraction data sets were collected at the beamline BL41XU in SPring-8 (Hyogo, Japan), and they were processed with the program HKL2000 [31] and the CCP4 program suite [32]. The crystal of the FAAP20-UBZ•K63-Ub_2_ complex belongs to the space group *P*2_1_, with unit cell dimensions of *a* = 59.8 Å, *b* = 45.9 Å, *c* = 172.9 Å, *β* = 98.1°. The complex structures were determined by molecular replacement using the program MolRep [33]. The crystal structure of Ub (PDB code: 1ubq) was used as the search model. The atomic model of FAAP20-UBZ was built to fit 2*F*_o_-*F*_c_ electron density map using the program COOT [34] with careful inspection. Refinement was carried out using the program Refmac5 [33] with iterative correction and refinement of the atomic model. The final model has excellent stereochemistry with an *R*_free_ value of 23.5% at 1.90 Å resolution. Data collection and refinement statistics are shown in S1 Table. All molecular graphics were prepared using the program PyMOL (DeLano Scientific; http://www.pymol.org).

### SPR analysis

GST-fused Ub and M1-Ub_2_ for SPR analyses were purified by Glutathione Sepharose FF and ResourceQ anion exchange columns (GE Healthcare). GST-fused K48-Ub_2_ was synthesized from Ub with an additional aspartate residue at its C-terminal extremity (D77 Ub) and GST-fused Ub carrying the K48R mutation. GST-fused K63-Ub_2_ was synthesized from D77 Ub and GST-fused K63R Ub. Ub conjugation reactions were performed as described previously [4]. Site-directed FAAP20-UBZ mutants were purified similarly to wild-type FAAP20-UBZ (see above). Experiments were carried out on a Biacore T200 instrument (GE Healthcare) equilibrated at 25°C in HBS-P buffer including 10 mM HEPES-Na (pH 7.4), 150 mM NaCl and 0.05% surfactant P 20, using a Sensor Chip C1 (GE Healthcare). Anti-GST antibodies (GE Healthcare) were covalently immobilized on the sensor chip at a density of about 1,800 resonance units (RU), and GST-fused Ub and Ub_2_ were captured on the sensor chip at a density of 200–400 RU. FAAP20-UBZ proteins were then injected for 60 sec at a flow rate of 10 μL∕ min. Equilibrium dissociation constants (*K*_d_) were computed by fitting to a 1:1 interaction model using Biacore T200 evaluation software (GE Healthcare). All assays were carried out three times for each sample. The data are presented as means ± SD.

### GST pull-down assays

GST-fused FAAP20-UBZs and FAAP20 were produced and purified similarly to FAAP20-UBZ, without the HRV3C protease digestion step. GST, GST-fused FAAP20 and GST-fused FAAP20-UBZ were immobilized on Glutathione Sepharose FF beads pre-equilibrated in a pull-down buffer (25 mM Tris-HCl [pH 8.0], 1 mM DTT, 0.1% Triton, 100 mM NaCl) and then incubated with K63-, K48- or linear Ub_2_ for 15 min at 4°C on ice in the pull-down buffer. The beads were extensively washed with the same buffer thrice. The diubiquitin molecules bound to the beads were released by incubating in SDS loading buffer without boiling, analyzed by SDS–PAGE and stained by Coomassie brilliant blue.

### Protein recruitment to laser-induced localized ICLs

GM0637 cells [35] (a simian virus 40-transformed human fibroblast cell line) were seeded in a 35 mm glass bottom culture dish, and they were incubated with 6 μM trioxalen at 37°C for 20 min prior to laser treatment. Thereafter, the DMEM was replaced by Leivobitz's L-15 (Gibco) containing 10% FBS and 25 mM HEPES (Gibco) and incubated at 37°C. After 10 min, the glass bottom culture dish was mounted on the microscope stage and maintained at 37°C throughout an experiment. UVA microirradiation experiment and live-cell imaging analysis were performed using a ZEISS LSM780 confocal laser-scanning microscope with a C-apochromat 63x /1.20 W Korr M27 objective. The 355 nm line of laser-UVA was used for microirradiation (three pulses at 520 μW), and images were acquired before and at 10 min after microirradiation. Cells were fixed immediately after image acquisition of GFP signals at 10 min after microirradiation with PBS containing 4% paraformaldehyde for 10 min. Thereafter, cells were permeabilized with PBS containing 0.1% SDS and 0.5% Triton X-100 for 10 min at room temperature. Cells were then incubated with mouse anti-γH2AX antibodies (1:20,000; Upstate) in PBS containing 1% BSA at 37°C for 30 min. Alexa Fluo 594-conjugated goat anti-mouse (1:2,000; Molecular Probes) was used as the secondary antibody. Nuclei were stained with DAPI. Samples were examined with a ZEISS Axioplan2 microscope controlled by Axiovision. At least 20 cells were microirradiated and subjected to immunofluorescence microscopic analyses in each experiment.

## Results and Discussion

### Overall structure of FAAP20-UBZ in complex with K63-linked Ub_2_

**Fig 1:**
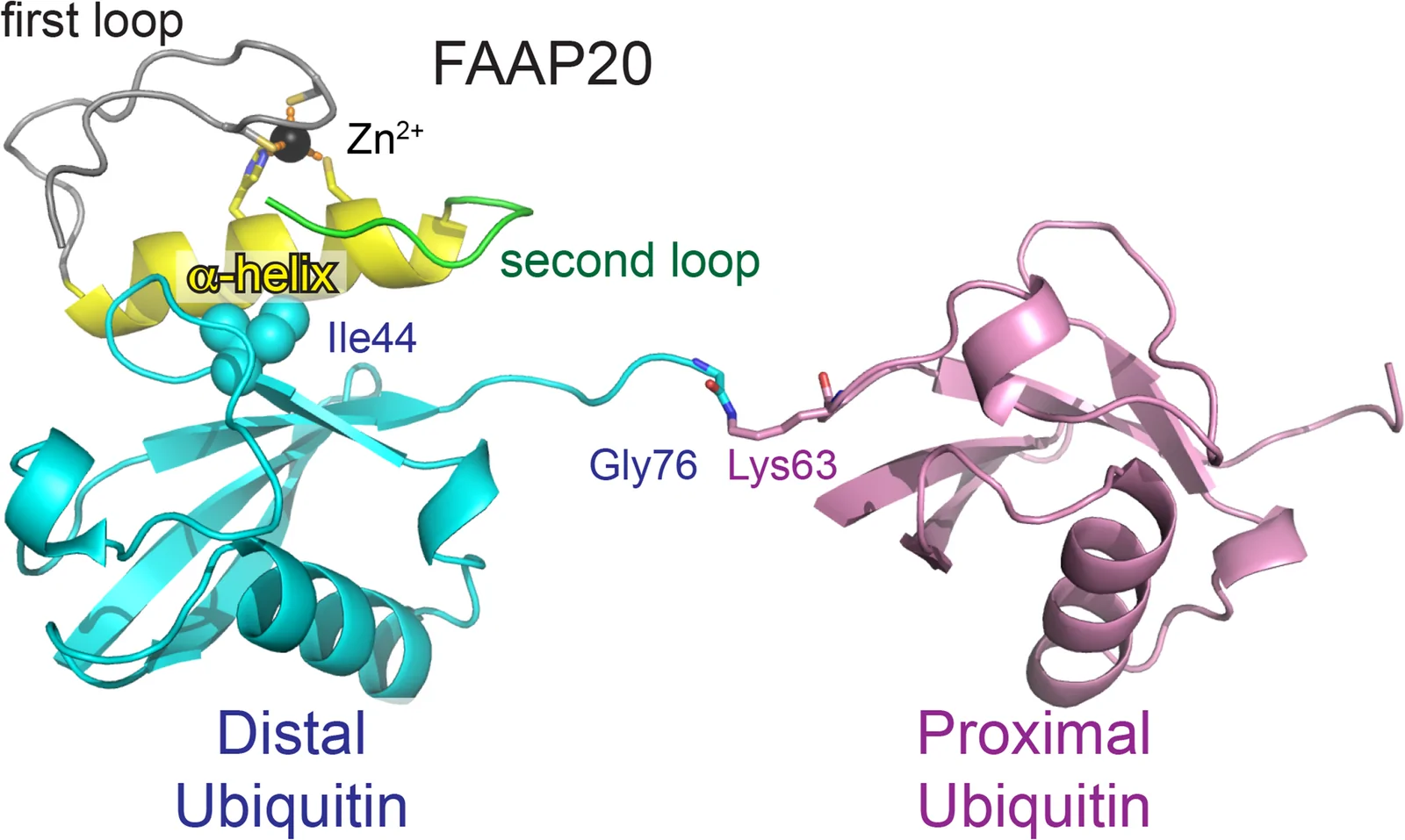
Crystal structure of FAAP20-UBZ in complex with K63-Ub_2_. Zinc-coordinating residues (Cys147, Cys150, Cys166 and His169) of FAAP20-UBZ and the isopeptide linkage (Gly76[Ub^distal^]–Lys63[Ub^proximal^]) are shown as sticks. The proximal and distal Ub moieties are colored pink and cyan, respectively. The first and second loops of FAAP20-UBZ are colored gray and green respectively. The central α-helix of FAAP20-UBZ is colored yellow. Ile44 of the distal Ub is shown as spheres. The coordinated zinc ion is shown as a black sphere. Hydrogen bonds are indicated as dashed orange lines.

FAAP20 is a 20 kDa protein, which binds to FANCA through its N-terminal region and contains a conserved UBZ domain in its C terminal extremity. To elucidate the mechanism underlying the specific interaction between FAAP20-UBZ and Ub chains, we determined the crystal structure of FAAP20-UBZ (residues 143–180) in complex with K63-linked Ub_2_ (K63-Ub_2_) at 1.9 Å resolution (S1 Table). Unlike other UBZ structures determined so far, FAAP20-UBZ lacks the two short β strands found in the classical ββα-fold (Fig. 1) [26–29]. The central α-helix (residues 160–173) is flanked between two loops (residues 143–159 and 174–180, respectively). A zinc ion is coordinated by the conserved residues Cys147, Cys150, His166 and Cys170 and bridges the first loop and the α-helix. Both the α-helix and the second loop interact with the distal Ub of K63-Ub_2_. In contrast, the proximal Ub does not interact with FAAP20 but contact two distinct neighboring proximal ubiquitins in the crystal through their Ile44 patches (S1 Fig.). Lys63 of one distal ubiquitin could not be linked to Gly76 of its neighboring ubiquitins. The distances between Leu71 of one distal ubiquitin and Lys63 of its neighboring ubiquitins are 34.0 Å and 34.8 Å, which are much longer than the length of the flexible C-terminal region of ubiquitin (Leu71-Gly76) that links two ubiquitins (16.4 Å). Consequently, FAAP20-UBZ interacts with one Ub moiety of K63-Ub_2_ in the present structure of the FAAP20-UBZ•K63-Ub_2_ complex, suggesting that FAAP20-UBZ can bind Ub as well as K63 and other chains.

To assess this possibility, we measured the affinity of FAAP20-UBZ with Ub or K63-, K48- or M1-linked Ub_2_ using surface plasmon resonance (SPR) spectroscopy (Table 1 and S2 Fig.). FAAP20-UBZ binds Ub efficiently (*K*_d_ = 19.9 μM). The equilibrium dissociation constant is lower than those of other UBDs [36]. In particular, the binding affinity of FAAP20-UBZ for Ub seems higher than that of the Polη UBZ (*K*_d_ = 81 μM, measured by isothermal titration calorimetry) [26]. The equilibrium dissociation constants of FAAP20-UBZ for the Ub_2_ species are either equivalent (M1) or twice higher (K63 and K48) than that for Ub. Therefore, FAAP20-UBZ can bind Ub and has little linkage specificity towards M1-, K48- and K63-linked Ub_2_ species. In the FAAP20-UBZ•K63-Ub_2_ structure, one FAAP20-UBZ molecule interacts with one K63-Ub_2_ molecule. This 1:1 stoichiometry may be an artifact of crystallization, because, in our SPR experiment, the actual *R*_max_ for binding of FAAP20-UBZ to K63-Ub_2_ is 30.0 ± 2.2 resonance units (RU), which is significantly higher than the theoretical *R*_max_ calculated from a 1:1 stoichiometry model (18.8 ± 0.4 RU) and slightly lower than that from a 2:1 (UBZ:Ub_2_) model (37.6 ± 0.9 RU). Therefore, two FAAP20-UBZ molecules can simultaneously bind the two Ub moieties of K63-Ub_2_ in solution.

**Table 1:** Equilibrium dissociation constants of FAAP20-UBZ for Ub and Ub_2_.

	***K***_*d*_ (μM)*	**Fold of increase**
Ub	19.9 ± 3.9	1.0
M1-linked Ub_2_	22.3 ± 1.3	1.1
K48-linked Ub_2_	39.1 ± 5.1	2.0
K63-linked Ub_2_	35.9 ± 4.4	1.8

* Values correspond to means of triplicate measurements ± standard deviation.

In addition, we tested the interaction of FAAP20 with K63-, K48- or M1-linked Ub_2_ by GST-pulldown assay. As shown in S3 Fig., both the full-length FAAP20 (1–180) and FAAP20-UBZ (142–180) can similarly bind these three different diubiquitin chains. Altogether our data indicate that FAAP20 does not display any Ub chain specificity in apparent contradiction with two previous publications, which suggested that FAAP20-UBZ preferentially binds K63 chains over K48 chains [11, 13]. Both relied on GST-pull down experiments, where the input ubiquitin chains had different length between K63 and K48-linked chains. In contrast, pull-down experiments using K63 and K48 chains of similar length did not show any difference [12], consistent with our data. However, we cannot exclude the possibility that other subunit(s) in the FA core complex might confer K63 specificity to FAAP20-UBZ. FANCA could be the candidate, because it directly binds to FAAP20.

### Interactions between FAAP20-UBZ and Ub

**Fig 2:**
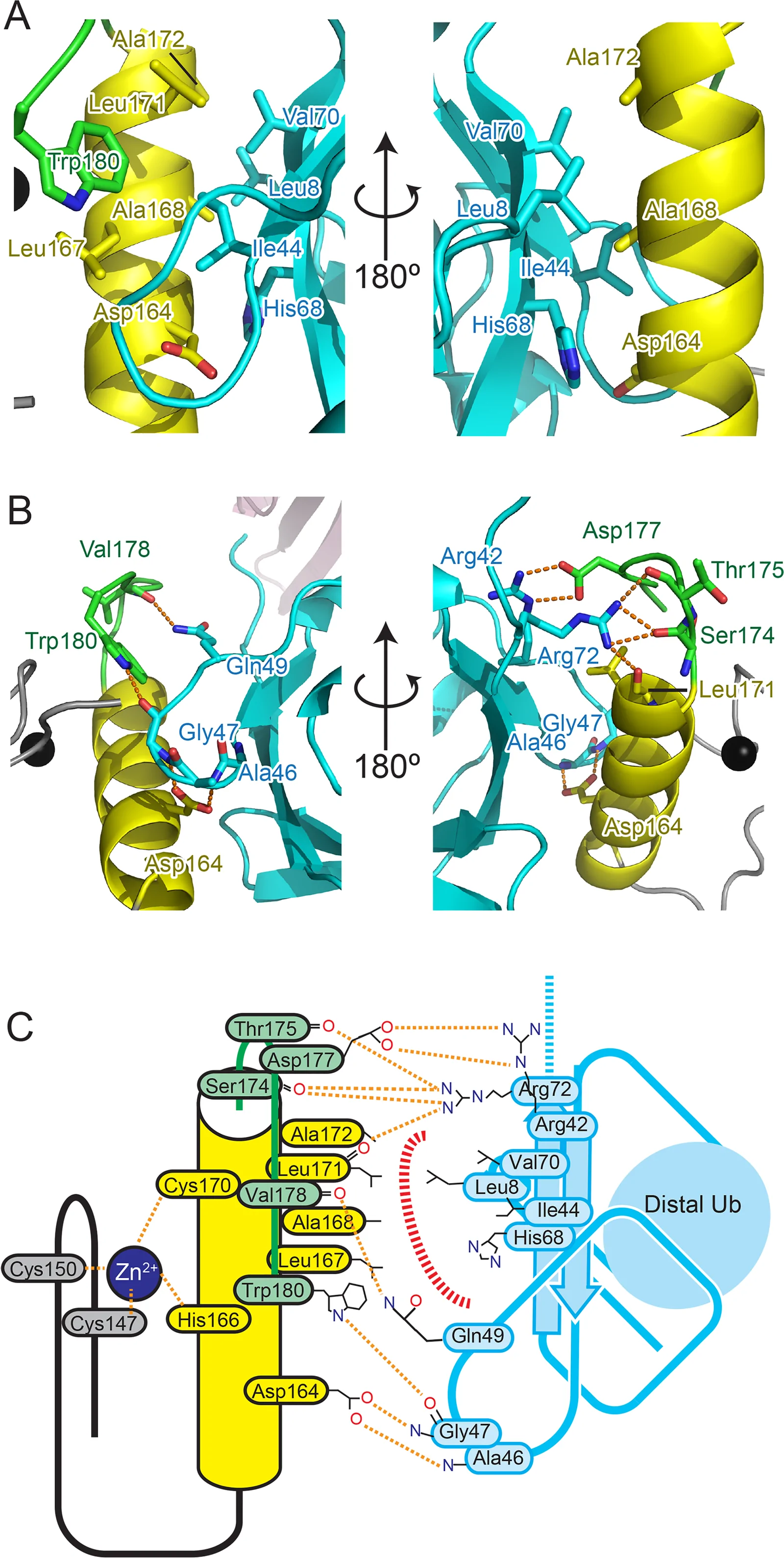
Ub recognition by FAAP20-UBZ. The coloring scheme is the same as that in Fig. 1. (A) Hydrophobic interactions between FAAP20-UBZ and Ub. (B) Hydrogen bond network between FAAP20-UBZ and Ub. (C) Schematic representation of the interface between FAAP20-UBZ and Ub. Hydrophobic interactions are displayed as dashed red lines.

Both hydrophobic interactions and hydrogen bonds constitute the interaction between FAAP20-UBZ and Ub. At the UBZ α helix, the side chains of Leu161, Leu167, Ala168, Leu171 and Ala172 and the aliphatic portion of Asp164 hydrophobically interact with the canonical Ile44-centered hydrophobic patch of Ub, which is formed by the side chains of Leu8, Ile44 and Val70 and the aliphatic portions of Thr66 and His68 side chains (Fig. 2A). The Asp164 Oδ atoms of FAAP20-UBZ further hydrogen bond with the main-chain amide groups of Ala46 and Gly47 of Ub, respectively. Trp180, which is located at the C-terminal extremity of the second loop of FAAP20-UBZ, also participates in the hydrophobic interaction with Ile44 of Ub. In addition, its Nε atom hydrogen bonds with the main-chain CO group of Gly47 of Ub (Fig. 2B). At the C-terminal part of the UBZ α-helix, the main-chain CO group of Leu171 hydrogen bonds with the Nη atom of Ub Arg72. The following second loop of FAAP20-UBZ also interacts with Arg72 of Ub. The main-chain CO groups of Ser174 and Thr175 in FAAP20-UBZ hydrogen bond with the two Nη atoms of Arg72 in Ub, respectively. Moreover, the two Oδ atoms of Asp177 on the second loop hydrogen bond with Nε and Nη of Arg42 in Ub, respectively, and the main-chain CO group of Val178 hydrogen bonds with Nε of Gln49 in Ub.

Contribution of these intermolecular interactions to Ub binding was examined by SPR analysis of site-directed FAAP20-UBZ mutants (Table 2 and S4 Fig.). No binding could be measured with mutations D164A, A168L and W180A, similarly to a mutation of a zinc-coordinating residue Cys150, which disables the bridging between the first loop and the central α-helix and thus likely destabilizes the FAAP20-UBZ structure. These results indicate that the central core interactions are essential for Ub binding. On the other hand, binding of L161A, A172L and D177A mutants was significantly reduced but could be measured. L161A, A172L and D177A mutations decreased the affinities 6.3-, 5.7- and 10.9-fold, respectively. Therefore, Ub binding of FAAP20-UBZ relies primarily on the central core interactions, which is enhanced by their surrounding interactions.

**Table 2:** Equilibrium dissociation constants of FAAP20-UBZ mutants for Ub.

	***K***_*d*_ (μM)*	**Fold of increase**
WT	19.9 ± 3.9	1.0
C150S	ND^†^	
L161A	125.0 ± 9.8	6.3
D164A	ND^†^	
A168L	ND^†^	
A172L	112.8 ± 14.1	5.7
D177A	217.5 ± 15.0	10.9
W180A	ND^†^	

*Values correspond to means of triplicate measurements ± standard deviation.

^†^ ND indicates no ubiquitin binding measured, corresponding to an equilibrium dissociation constant higher than 300 μM.

### In vivo accumulation of FAAP20 to ICL sites, depending on the UBZ–Ub interaction

**Fig 3:**
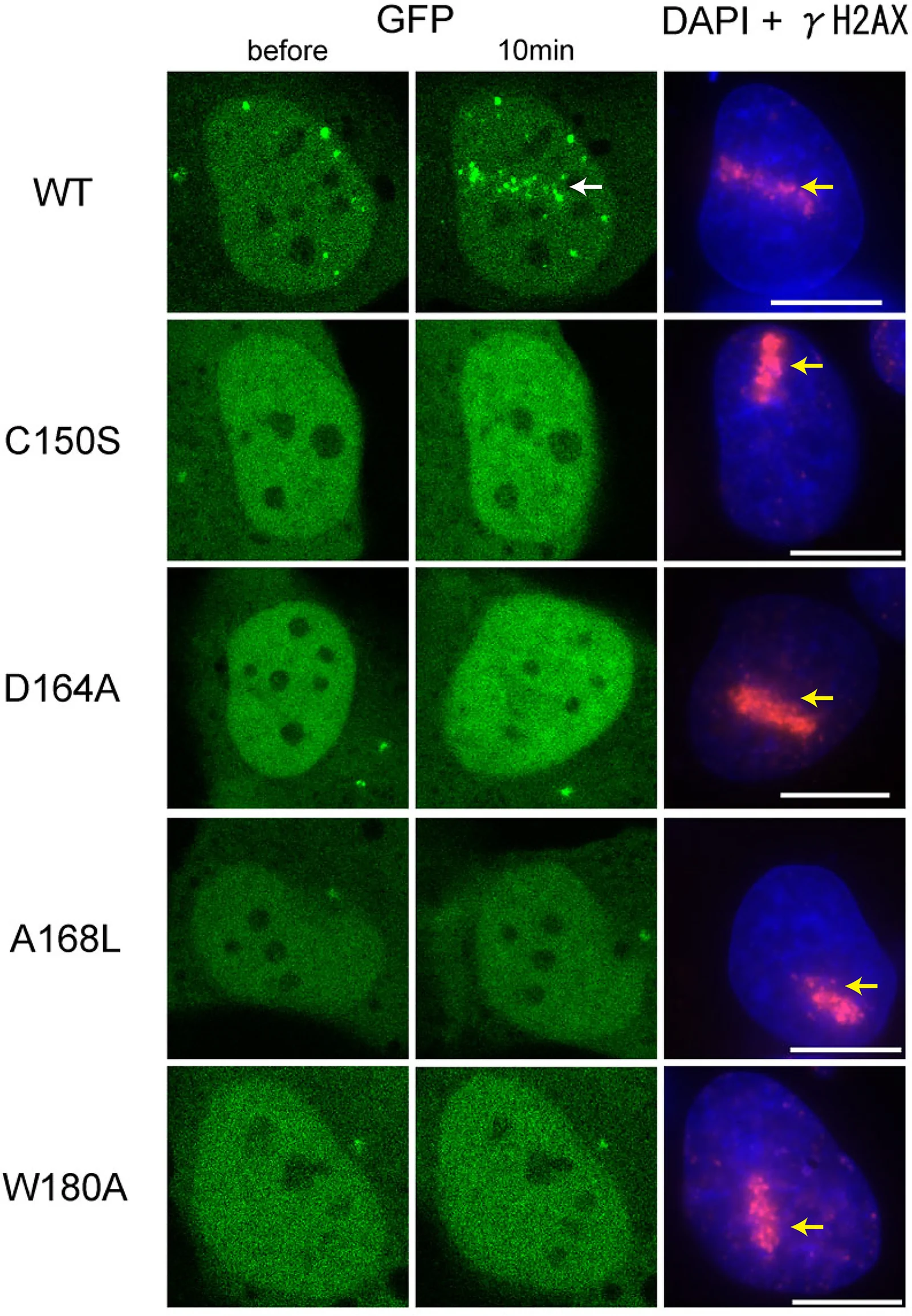
Recruitment of wild-type and mutant FAAP20 to ICLs. GFP-tagged FAAP20 proteins were detected before and 10 min after laser induced ICLs formation, by confocal imaging. The white and yellow arrows indicate the accumulation of FAAP20 and the γH2AX-positive DNA damage sites, respectively. Scale bars: 10 μm.

After ICL formation, FAAP20 is recruited to DNA damage sites. This localization requires a functional UBZ domain [11, 13, 25]. We tested if FAAP20 proteins bearing the aforementioned mutations were still able to localize to ICL sites. We generated ICLs by irradiating cells with UVA after pre-incubation with psoralen and monitored accumulation of GFP-tagged FAAP20 to DNA damage sites by confocal imaging (Fig. 3). Wild-type FAAP20 was accumulated to ICL sites 10 minutes after psoralen-UVA treatment, as shown in previous studies [12]. In contrast, C150S, D164A, A168L and W180A mutants adopted a diffuse localization throughout the cells. Therefore, the residues that are essential for Ub binding *in vitro* are also required for proper recruitment of FAAP20 to DNA damage sites *in vivo*. These results also ensure that binding between FAAP20-UBZ and Ub is physiologically essential for the recruitment of FAAP20 to DNA damage sites.

### Comparison of FAAP20-UBZ with WRNIP1-, RAD18-, Polη- and NEMO-UBZ

**Fig 4:**
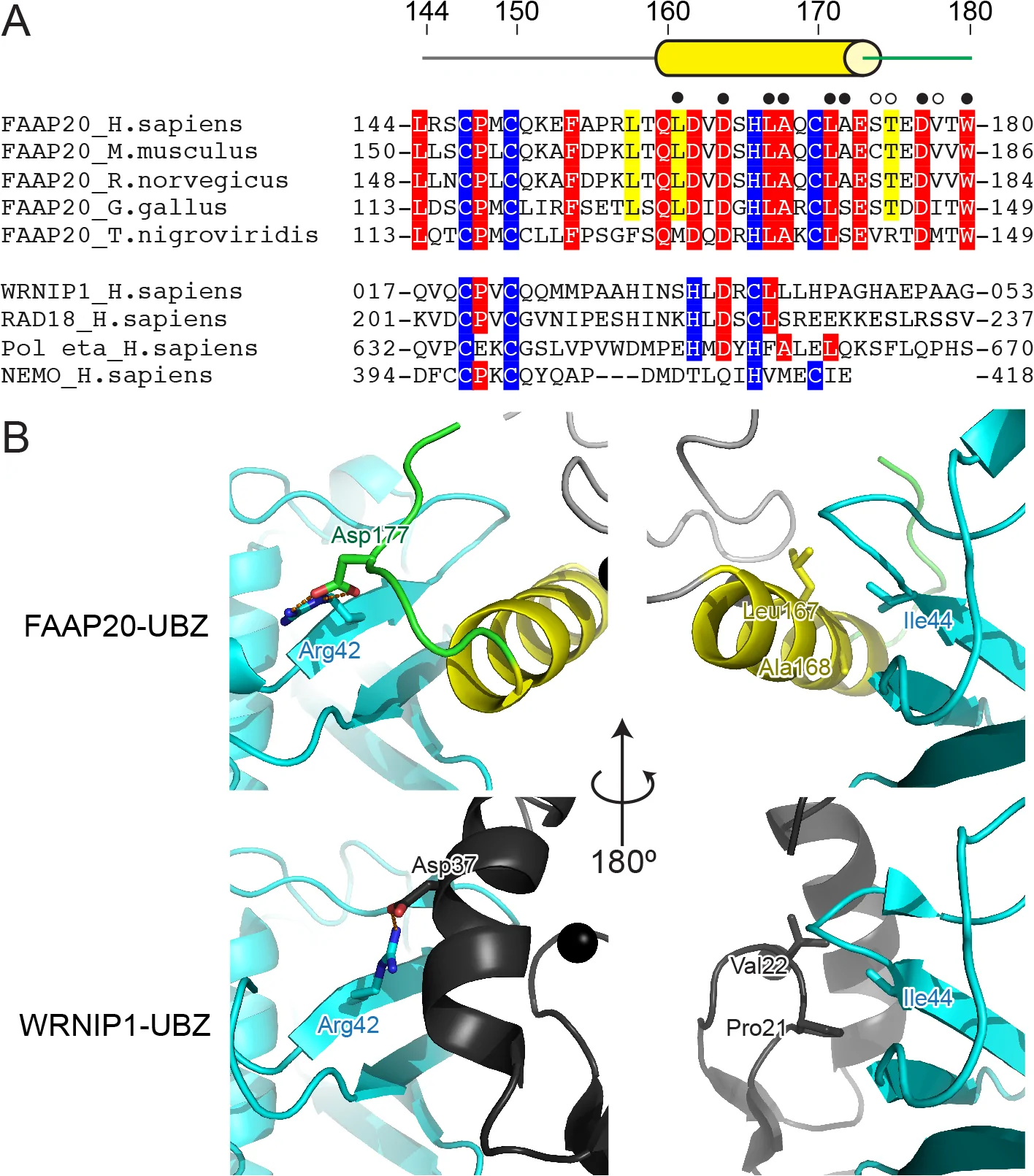
Amino-acid sequence comparison of Polη-, NEMO- and FAAP20-UBZs. (A) Sequence alignment of five vertebrate FAAP20-UBZs (human, mouse, rat, chicken and puffy fish) and human WRNIP1, RAD18, Polη- and NEMO-UBZs. 100% and more than 80% identical residues among the FAAP20 protein are highlighted by red and yellow background, respectively. Zinc-ion-coordinating residues are highlighted in blue. Open or filled circles indicate the residues that interact with Ub through their main or side chains, respectively (B) Structural comparison of FAAP20- with WRNIP1-UBZ. The coloring scheme is the same as that in Fig. 1. WRNIP1-UBZs are colored dark gray.

The Ub-interacting residues of FAAP20-UBZ are conserved or replaced by functionally equivalent residues among human, mouse, rat, chicken and puffy fish (Fig. 4A). On the other hand, we could not assess the conservation of these residues among other UBZs, due to their highly divergent amino-acid sequences. Three-dimensional structural information is required to correctly align their sequences.

To date, UBZ structures of WRNIP1 and RAD18 have been solved in complex with Ub [29]. The Ub-interacting residues are conserved between both proteins [29]. WRNIP1-UBZ (PDB code: 3VHT) and RAD18-UBZ (PDB code: 2MRE) display the canonical ββα fold, in which the loop between both antiparallel β-strands (β-loop) and the α-helix interact with Ub. As shown in Fig. 4B, the central α-helix of WRNIP1 occupies a position similar to the C-terminal loop of FAAP20. Arg42 of Ub interacts with Asp37 in the α-helix of WRNIP1 and Asp177 in the C-terminal loop of FAAP20. Similarly, the β-loop of WRNIP1 occupies a part of the FAAP20 α-helix position. In particular, Ile44 of Ub interacts with Pro21 and Val22 in the β-loop, in place of Ala168 and Leu167 in the α-helix of FAAP20.

**Fig 5:**
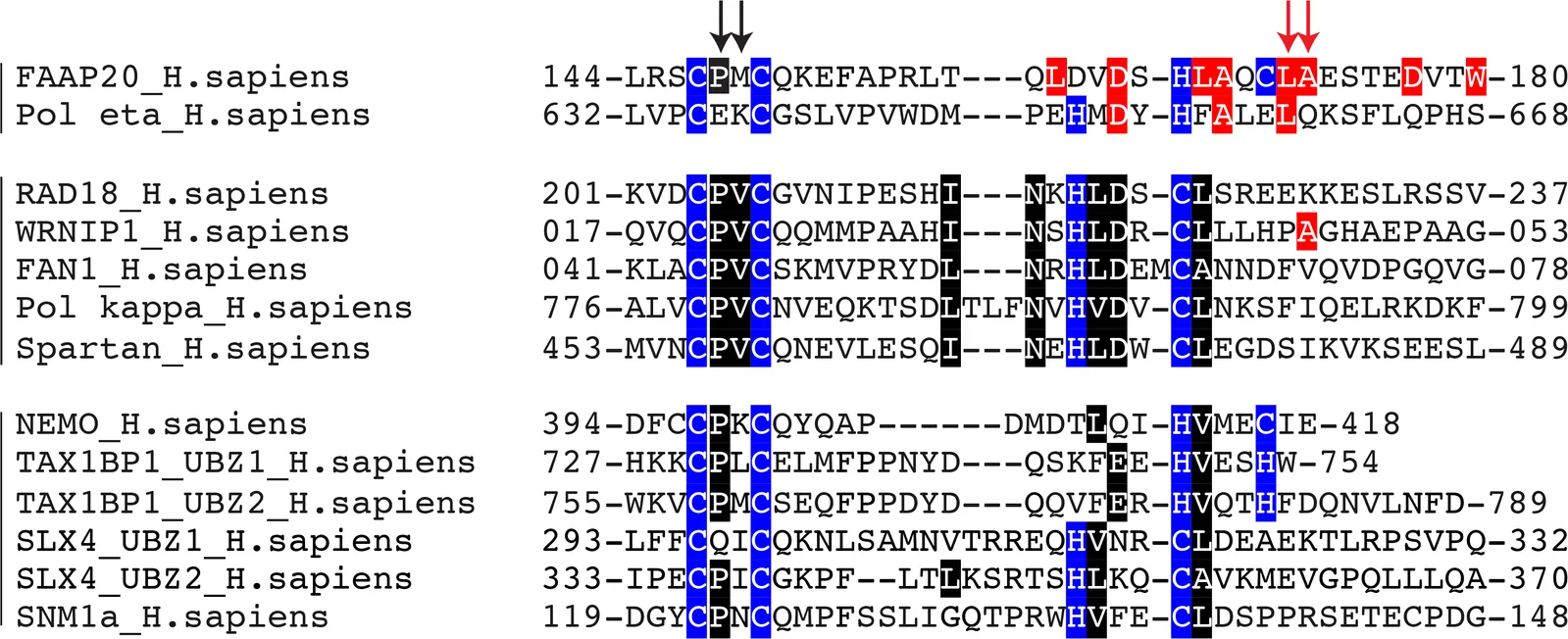
New classification of UBZ family proteins. The sequence of UBZs were aligned using ClustalW2. The sequences of NEMO- and Polη-UBZs were aligned, based on their three-dimensional structures. The sequences were then divided according to the presence of Ub-binding residues. The Ub-binding residues related to FAAP20-UBZ are highlighted in red, while those related to WRNIP1-UBZ and Rad18-UBZ are highlighted in black. The zinc-ion-coordinating residues are highlighted in blue. Black and red arrows indicate the positions of WRNIP1 Pro21 and Val22 and FAAP20 Leu167 and Ala168, respectively.

Next, we analyzed other members of the UBZ family. UBZ structures of Polη and NEMO (Polη- and NEMO-UBZ, respectively) have been solved in isolated forms [26, 27]. Both NEMO-UBZ (PDB code: 2JVX) and Polη-UBZ (PDB code: 2I5O) seem to interact with Ub in a manner different from RAD18-UBZ and WRNIP1-UBZ. The highly conserved Pro21-Val22 in the β-loop of WRNIP1 are replaced by Pro398-Lys399 in NEMO-UBZ and Glu636-Lys637 in Polη (Fig. 4A). The presence of charged residues should disrupt the hydrophobic interaction with Ile44. In contrast, four Ub-interacting residues of FAAP20-UBZ (*i*.*e*., Asp164, Leu167, Ala168 and Leu171) are conserved or replaced by a functionally equivalent residue in Polη-UBZ (*i*.*e*., Asp652, Phe655, Ala656 and Leu659) (Fig. 4A and S5 Fig.). This sequence conservation suggests that Polη-UBZ binds Ub in a similar manner to FAAP20-UBZ. On the other hand, these residues are poorly conserved in NEMO-UBZ, where Asp164, Leu167, Ala168 and Leu171 of FAAP20-UBZ are replaced by Gln411, Val414, Met415 and Ile418, respectively. Importantly, the side chain of Met415 of NEMO-UBZ is much larger than that of Ala168 of FAAP20-UBZ and thus cannot fit in the Ile44-centered hydrophobic patch of Ub, suggesting a different mode of interaction with Ub (Fig. 4 and S5 Fig.). The alanine residue corresponding to Ala168 of FAAP20-UBZ seems also missing in other UBZs including WRNIP1 and RAD18 (Fig. 5).

Altogether, our data suggest the presence of at least three groups of UBZ domains (Fig. 5). In the FAAP20/Polη group, the β-loop is absent or too divergent, so that Ile44 interacts with hydrophobic residues in the α-helix. The WRNIP1/Rad18 group interacts with Ile44 of Ub through their β-loop. Finally, the third group of UBZ domains comprises proteins that possess neither the FAAP20-UBZ residues involved in Ub-binding (highlighted in red) nor the RAD18-UBZ residues involved in Ub-binding (highlighted in black), and their mode of interaction should be different from the two other groups. The UBZ domains have been classified, according to the zinc coordinating residues (namely type 3 for CCHH and type 4 for CCHC). However, this classification seems not appropriate for representing the structural information, since several type-3 UBZs interact differently with Ub (NEMO-UBZ, FAAP20-UBZ and WRNIP1-UBZ).

### Comparison of FAAP20-UBZ with other UBDs

**Fig 6:**
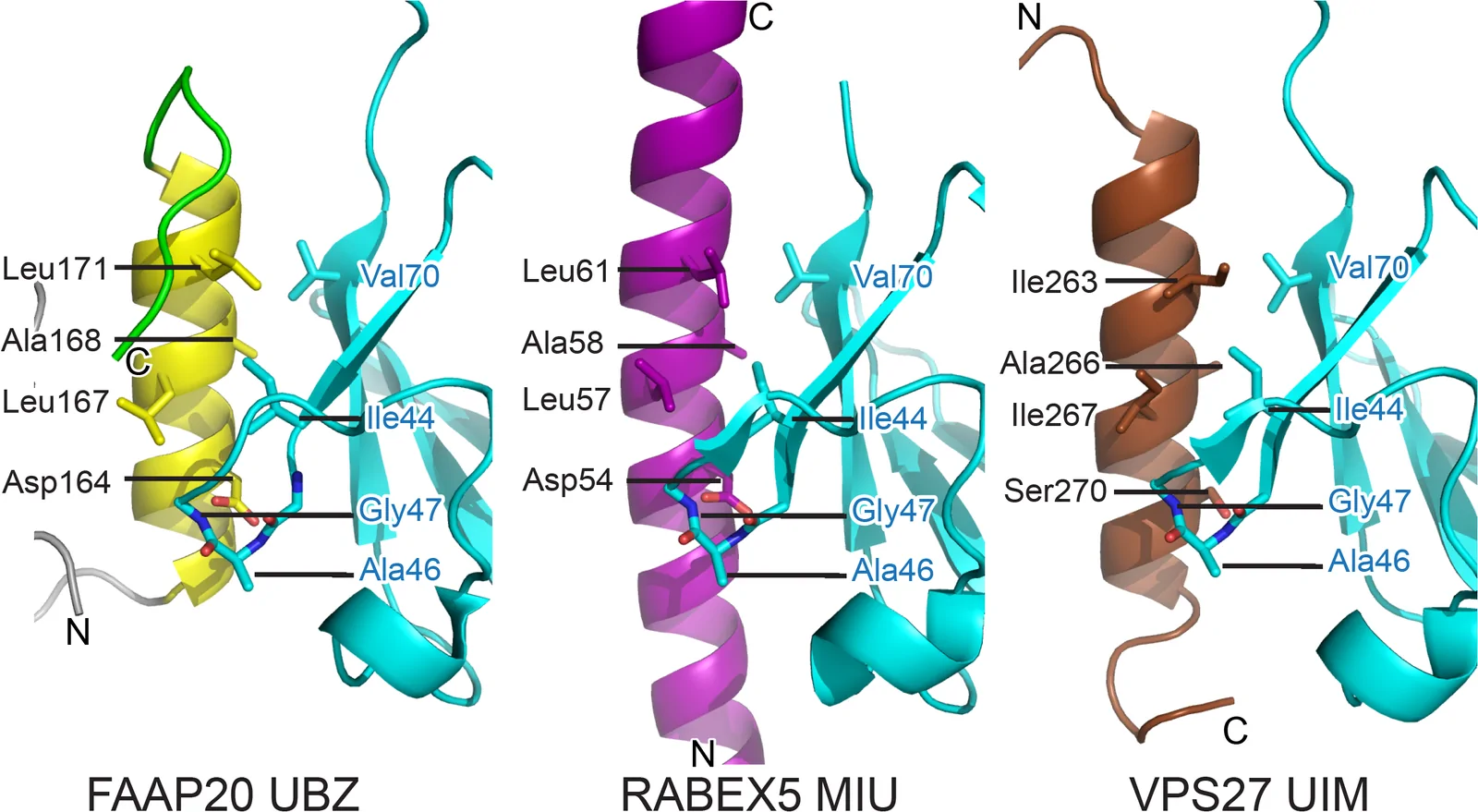
Structural comparison of FAAP20-UBZ, RABEX-MIU and VPS27-UIM. The coloring scheme is the same as that in Fig. 1. RABEX-MIU and VPS27-UIM are colored purple and brown, respectively.

To further investigate whether the UBZ-Ub interface resembles other UBD-Ub interface, we compared the Ub-interacting α-helix of FAAP20-UBZ with Ub-binding single α-helix motifs such as UIM (Ub-interacting motif) and MIU (motif interacting with Ub, also known as IUIM [inverted UIM]) (Fig. 6). Three-dimensional structures of the UIM domain of Vps27 (Vps27-UIM) (PDB code: 1Q0W) and the MIU domain of Rabex5 (Rabex5-MIU) (PDB code: 2C7M and 2FIF) have been solved in complex with Ub [37–39]. Rabex5-MIU and FAAP20-UBZ are orientated in the same direction, relative to the bound Ub. Remarkably, critical residues involved in Ub binding are strictly identical between Rabex5-MIU and FAAP20-UBZ. Thus, the α-helix-mediated Ub-interaction of FAAP20-UBZ is closely related to that of MIU. Although the UIM polarity towards the bound Ub is opposite to FAAP20-UBZ and Rabex5-MIU, most of the Ub-interacting residues on the α-helix are identical or functionally equivalent among Vps27-UIM, FAAP20-UBZ and Rabex5-MIU, suggesting convergent evolution of these three distinct Ub-binding motifs.

## Conclusion

In this study, we determined the crystal structure of FAAP20-UBZ in complex with K63-Ub_2_. Analyses of site-directed FAAP20-UBZ mutants *in vitro* and *in vivo* support the relevance of the observed physical interactions. Taken together, our results indicate that FAAP20-UBZ binds Ub and Ub chains with similar affinities. FAAP20 may be recruited by polyubiquitylated histones, as previous studies on FAAP20 suggested [11–13], but the absence of K63 specificity suggests that additional targets, including mono-ubiquitylated proteins, can be involved in recruiting FAAP20 to DNA damage location. Further investigation is required to understand the precise recruitment and function of FAAP20. Ub-interacting mode of FAAP20-UBZ differs from that of WRNIP1- and RAD18-UBZ. We propose a new classification of UBZ domains, according to their mode of interaction with Ub: The first group includes FAAP20 and Polη, while the second group includes WRNIP1 and RAD18. There is a third group that lacks Ub-interacting residues conserved in the first or second UBZ group. Solving the structure of the third group would be necessary to get a full comprehension of Ub recognition by UBZ-family members.

## Supporting Information

S1 Fig Four FAAP20-UBZ•K63-Ub_2_ complexes in the asymmetric unit. The proximal and distal Ub moieties are colored pink and cyan, respectively. The first and second loops of FAAP20-UBZ are colored gray and green, respectively. The central α-helix of FAAP20-UBZ is colored in yellow.
(EPS)

S2 Fig SPR sensorgrams for analyzing the FAAP20-UBZ(wt)–Ub and FAAP20-UBZ(wt)–Ub_2_ interactions. (EPS)

S3 Fig Pull-down assays using GST-fused full-length FAAP20 (1–180), FAAP20-UBZ (142–180) or GST only for binding to K63-, K48- or linear Ub_2_. (TIF)

S4 Fig SPR sensorgrams for analyzing the FAAP20-UBZ–Ub interaction using mutant or wt FAAP20-UBZ. (EPS)

S5 Fig Structural comparison of FAAP20-, Polη- and NEMO-UBZs. The coloring scheme is the same as that in Fig. 1. Polη- and NEMO-UBZs are colored light orange and light green, respectively.
(EPS)

S1 Table Data collection and refinement statistics. The numbers in parentheses are for the highest resolution shell. *R*_*sym*_ = ∑|*I*_*avg*_ − *I*_*i*_|/∑*I*_*i*_, *R*_*work*_ = ∑|*F*_*obs*_ − *F*_*calc*_|/∑*F*_*obs*_ for reflections of working set, *R*_*free*_ = ∑|*F*_*obs*_ − *F*_*calc*_|/∑*F*_*obs*_ for reflections of test set (5% of total unique reflections).
(DOCX)
