# Supplementary figures and images for: Structural Basis for Ubiquitin Recognition by Ubiquitin-Binding Zinc Finger of FAAP20
Source: PLoS One. 2015 Mar 23;10(3):e0120887. doi: 10.1371/journal.pone.0120887 (PMC4370504; doi:10.1371/journal.pone.0120887)

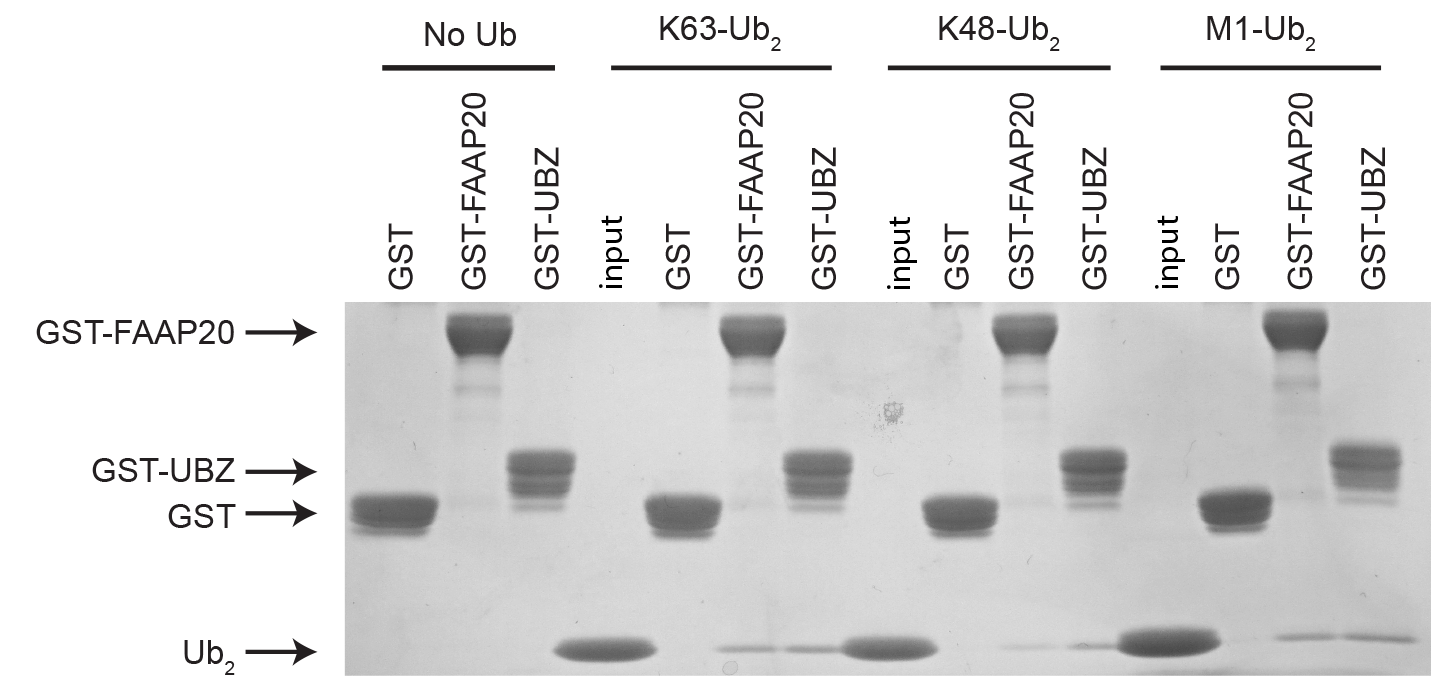

Supplement: S3 Fig — (TIF) [file pone.0120887.s003.tif]
