# Supplementary material for: Structural Basis for Ubiquitin Recognition by Ubiquitin-Binding Zinc Finger of FAAP20
Source: PLoS One. 2015 Mar 23;10(3):e0120887. doi: 10.1371/journal.pone.0120887 (PMC4370504; doi:10.1371/journal.pone.0120887)
Supplement: S1 Table — R sym = ∑|I avg − I i|/∑I i, R work = ∑|F obs − F calc|/∑F obs for reflections of working set, R free = ∑|F obs − F calc|/∑F obs for reflections of test set (5% of total unique reflections). (DOCX) [file pone.0120887.s006.docx]

Table S1. Data collection and refinement statistics

|  | FAAP20-UBZ•K63-Ub_2_ |
| --- | --- |
| **Beam Line** | Spring8 BL41XU |
| **Data Collection** |  |
| Space group | P2_1_ |
| Unit cell parameter | a = 59.8 Å, b = 45.9 Å, c = 172.9 Å, β = 98.1° |
| Wavelength, Å | 1.00000 |
| Resolution, Å | 50.0-1.90 (1.93-1.90) |
| Unique reflections | 74 073 |
| Total reflections | 210 217 |
| Completeness, % | 95.1 (88.5) |
| *I/σ(I)* | 27.0 (4.26) |
| R_sym_ | 0.064 (0.322) |
| **Refinement Statistics** |  |
| Number of atoms: protein, zinc, water | 6009, 4, 346 |
| Rmsd bond length, Å | 0.014 |
| Rmsd bond angle, ° | 1.860 |
| Average B factors (Å^2^): protein, zinc, water | 40.7, 26.4, 40.2 |
| Residues in core region, % | 96.2 |
| Residues in additionally allowed region, % | 3.8 |
| Residues in generously allowed region, % | 0 |
| Residues in disallowed region, % | 0 |
| R_work_, R_free_ | 0.208, 0.235 |

The numbers in parentheses are for the highest resolution shell.

*R_sym_* = Σ|*I_avg_* - *I_i_*| / Σ*I_i_*

*R_work_*=Σ|*F_obs_* - *F_calc_*| / Σ*F_obs_* for reflections of working set

*R_free_*=Σ|*F_obs_* - *F_calc_*| / Σ*F_obs_* for reflections of test set (5% of total unique reflections).
